# Pilot Testing of Peak Alpha Frequency Stability During Repetitive Transcranial Magnetic Stimulation

**DOI:** 10.3389/fpsyt.2018.00605

**Published:** 2018-11-20

**Authors:** Nicholas J. Petrosino, Amin Zandvakili, Linda L. Carpenter, Noah S. Philip

**Affiliations:** ^1^Center for Neurorestoration and Neurotechnology, Providence VA Medical Center, Providence, RI, United States; ^2^Department of Psychiatry and Human Behavior, Butler Hospital, Alpert Medical School of Brown University, Providence, RI, United States

**Keywords:** posttraumatic stress disorder, major depressive disorder, transcranial magnetic stimulation, neurostimulation, electroencephalography, intrinsic alpha frequency

## Abstract

Over half of those diagnosed with post-traumatic stress disorder (PTSD) have comorbid major depressive disorder (MDD), and rates are even higher among military veterans. Transcranial magnetic stimulation (TMS) may be a safe and efficacious treatment for PTSD, both with and without comorbid MDD. Still, the mechanism of action of TMS is not fully understood, and it remains unclear which stimulation techniques (e.g., target regions, pulse strength/frequency, waveform) optimize treatment for these patients. Recent research indicated that a patient's unique individualized alpha frequency (IAF) may be used to guide brain stimulation treatment, and emerging data suggests that stimulation synchronized to the IAF may be efficacious for MDD. However, to our knowledge there are no studies to date that evaluate the stability of IAF over time in patients with comorbid PTSD and MDD. To this end, we used an eight-lead electroencephalography (EEG) system to record IAF before and after a course of TMS. Stimulation parameters were informed by prior studies of TMS for comorbid PTSD and MDD and included 5 Hz TMS to the left dorsolateral prefrontal cortex, at 120% of motor threshold, 3,000–4,000 pulses per session for up to 40 sessions. We tested whether IAF was changed with a course of TMS therapy and evaluated whether IAF predicted clinical outcomes. We observed no significant changes in IAF from baseline to post-treatment, and there was no relationship between IAF and clinical symptom change. These data demonstrate the stability of IAF with TMS and indicate its utility as a trait marker for future brain stimulation studies. This work does not support the use of IAF as predictor of clinical response to TMS as administered.

## Introduction

Repetitive transcranial magnetic stimulation (rTMS, hereafter simply TMS) has emerged as an important new treatment for pharmacoresistant major depressive disorder (MDD). While the concept of stimulating the brain with magnetic fields is not new, TMS was introduced into clinical use after two large sham-controlled studies in patients with MDD demonstrated efficacy (1, 2). Since that time, TMS research has expanded into other psychiatric domains, and in particular, post-traumatic stress disorder (PTSD). Indeed, a recent meta-analysis on the relatively smaller body of literature on TMS for PTSD (3) suggested efficacy. And for those patients with comorbid disorders, our group has shown 5 Hz TMS to significantly reduce both MDD and PTSD symptom burden (4, 5).

Finding new ways to successfully treat clinically common psychiatric comorbidities is an important next step in treatment development. Both MDD and PTSD cause debilitating symptoms and are associated with significant psychosocial dysfunction. MDD is regarded as a preeminent cause of disability worldwide, with an estimated 350 million people affected, and over 800,000 deaths by suicide every year (6). PTSD is also common, with up to 7% of the US population suffering from the disorder at some point in their life (7), and affecting up to 70% of U.S. military veterans (8). Furthermore, comorbidity of PTSD and MDD is quite high, with up to 50% of PTSD patients also carrying the diagnosis of MDD, and even greater rates observed in military samples (9, 10).

Unfortunately, currently available TMS treatments use a “one size fits all” approach to stimulation, which likely contributes to suboptimal efficacy rates. Electroencephalography (EEG) biomarkers show promise for improving the efficacy of TMS therapy based on the notion that its therapeutic mechanism of action is related to entrainment of oscillatory neuronal activity in the alpha (8–13 Hz) frequency band [(11); reviewed in (12)]. TMS treatments under development [e.g., (13)] are exploring the potential utility of using a personalized medicine approach which stimulates the cortex with pulses delivered at the patient's unique individual alpha frequency (IAF). This approach is supported by a body of literature which indicates that pathological oscillatory states are associated with depression, and thus potentially related to the mechanisms of action of TMS. From this perspective, MDD can be included in a group of disorders termed “thalamocortical dysrhythmias” (i.e., psychiatric illnesses that demonstrate abnormal rhythms associated with increased synchrony of neuronal oscillations across many brain regions primarily in the theta and alpha frequency bands) (14–19).

Therefore, it is possible that TMS is an effective treatment for MDD insofar as it interrupts or resets the hypersynchronizing, alpha-dominant thalamocortical oscillators via transient entrainment, allowing for the re-emergence of the more physiologically normal “intrinsic” oscillator activity and increasing neural plasticity (11, 20–22).

Yet this work is largely based on the assumption that measurable brain frequencies, and particularly the IAF, is a stable and useful measure of oscillatory activity [e.g., (23)]. Furthermore, some case report data suggest that IAF may also predict clinical response to TMS treatment (24). So, if future new brain stimulation approaches are designed to target the IAF–and be successful in reducing symptoms of depression in the context of commonly comorbid PTSD–then it is important to establish whether the IAF is indeed constant and whether it is associated with treatment response. To this end, we performed pilot testing of EEG data acquired as a part of a larger TMS study [(5); described in further detail below], hypothesizing that IAF would be stable (i.e., not modified) by TMS, while also exploring whether IAF might predict treatment response or be related to baseline clinical symptom severity.

## Materials and methods

### Subjects and TMS

Thirty-five participants were recruited from hospitals affiliated with the Alpert Medical School of Brown University (Providence VA Medical Center and Butler Hospital), in Providence, RI, USA. Written informed consent was given by all subjects for the study procedures and was approved by the corresponding Institutional Review Boards. Participants were adults ages 18–75 who met DSM-IV-TR (25) criteria for both PTSD and MDD with moderate severity of symptoms as confirmed by a psychiatrist using the Clinical Global Impressions (CGI) scale (26). Psychiatric medications were required to be unchanged for at least 6 weeks prior to participation. A more detailed description of inclusion/exclusion criteria can be found elsewhere (5). Clinical outcome data was collected at baseline and follow-up and included measurement of PTSD symptoms using the PTSD Checklist for DSM-5 (PCL-5) and MDD symptoms using the Inventory of Depressive Symptomatology Self-Report (IDSSR). PTSD outcomes were measured in terms of participants achieving a clinically meaningful reduction in PTSD symptoms (PCL score reduction of at least 10 points from baseline) and those who no longer met threshold criteria for PTSD (score < 33) (27). Depression outcomes were measured in terms of participants achieving clinical response (reduction of at least 50% from baseline IDSSR score) and remission (score < 15) (28). Symptom data for those subjects who completed baseline procedures and at least one TMS session were analyzed in an intent-to-treat, last-observation-carried forward fashion.

Subjects received up to 40 sessions of 5 Hz TMS treatment. This stimulation frequency was selected as part of research in our laboratory investigating its potential use in patients with comorbid PTSD and MDD, and previously found (in case series data) that 5 Hz could reduce symptoms in both domains (4). Stimulation was delivered at 120% of motor threshold to the left dorsolateral prefrontal cortex in 4-s trains with a 12-s intertrain interval for 3,000 pulses per session. Pulses were increased to 4,000 per session (with a reduced intertrain interval to 11-s) in subjects for whom no significant improvement in symptoms were observed by the 15th session. The parent study was registered at clinicaltrials.gov (5 Hz Repetitive transcranial magnetic stimulation for post-traumatic stress disorder comorbid with major depressive disorder; clinicaltrials.gov; NCT02273063).

### EEG acquisition

Resting-state, eyes-closed EEG data was recorded and digitized with eight surface electrodes in all subjects before and after TMS treatment. During each EEG recording session, subjects were instructed to lie down in a quiet room, avoid movements, keep eyes open for 1 min, closed for 8 min, and open again for 1 min. Using an electrode cap with a commercial eight-channel EEG device (ENOBIO8, Neuroelectrics, Cambridge, MA, USA), dry electrodes were placed over Fp1, Fp2, Fpz, F3, Fz, Cz, Pz, and Oz according to the 10–20 system, and referenced against two connected mastoid electrodes. This relatively sparse method for EEG acquisition was used in order to minimize setup time and patient burden. Using a low-pass (50 Hz) and high-pass filter (0.5 Hz), EEG was sampled at 500 Hz and digitized at 24-bit precision.

### EEG data analysis

EEG data were re-referenced on both Fpz-Oz [following (13)] and bipolar montages and identical EEG analyses were performed on each resulting data set. For the bipolar montage, amplitude subtraction into eight nearest-neighbor bipolar electrode pairs was performed. The data was then segmented into 2-s non-overlapping epochs. Epochs that were found to contain artifact (from patient movements, eye or muscle movements) or amplifier drift were removed by manual inspection (masked for responders' status.) Only eyes-closed data from those subjects with >120 s (or 60 2-s epochs) of usable EEG were used in the analysis, and all others were excluded. Because our hypothesis involved stability of IAF with TMS, also excluded were those subjects who did not have EEG recorded both before and after treatment, as well as those who had poor signal from at least one electrode (which would confound our nearest neighbor analyses, described below.) Power spectral density of artifact-free 2-s epochs was then calculated using a Welch Power Spectral Density estimate and a Hamming window with a 50% overlap.

We used two methods to calculate IAFs: (1) Alpha peak frequency (IAF-PF) was calculated for baseline and follow-up data by determining the peak power value within a frequency range of 8.3–12.7 Hz (with accuracy of ±0.5 Hz) for all electrodes in all subjects; (2) Alpha center of gravity (IAF-CoG) which is a power spectral density weighted mean frequency in alpha range calculated in the alpha range (8–13 Hz). The Alpha center of gravity is biased toward reporting values in the middle of the frequency range (around 10.5 Hz in this case) yet it is more accurate in assessing IAF when there are multiple peaks or no clear peak in the data. A paired-sample *t*-test was then performed on the calculated IAFs to determine any significant changes from baseline to follow-up. A second paired-sample *t*-test was also performed to determine any significant difference between the individuals' relative alpha power at baseline and follow-up. Lastly, analyses using Pearson correlation coefficients and corresponding *p*-values were done to evaluate whether baseline IAF predicted response to TMS treatment on depression or PTSD symptoms. All results were corrected for multiple comparisons using Bonferroni correction.

## Results

Twenty-one (60%) participants had usable EEG data and were included in further data analyses. They were 51 ± 9.7 years old, and eight (38.1%) were female, with mean baseline symptom severity scores of 53.0 ± 13.5 and 48.6 ± 11.7 for the PCL-5 and IDSSR, respectively. Fourteen (66.7%) participants showed meaningful clinical improvement on the PCL-5, and 11 (52.4%) showed a decrease to threshold for categorical PTSD response. For depressive symptoms as measured on the IDSSR, nine (42.9%) participants achieved clinical response and seven (33.3%) remitted. Symptom reductions measured on the PTSD and MDD scales were highly correlated (*r* = 0.91, *p* < 0.001).

**Figure 1:**
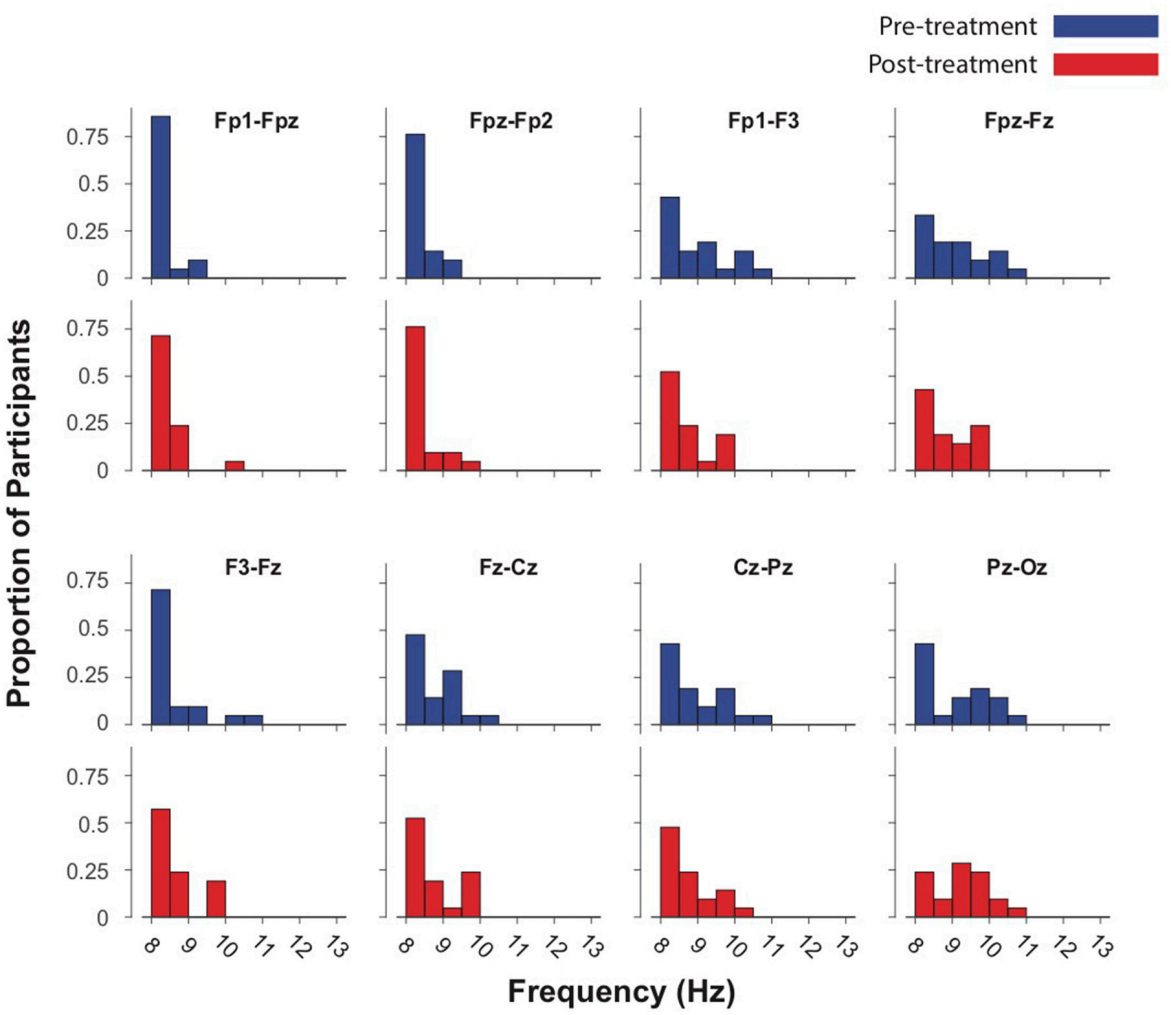
Intrinsic alpha frequencies (IAFs) of 21 subjects in eight EEG channels re-referenced to nearest-neighbor pre- and post-treatment (up to 40 sessions of 5 Hz TMS.) Blue bars represent baseline IAFs. Paired-sample *t*-tests demonstrated no significant difference in IAF before and after treatment.

**Figure 2:**
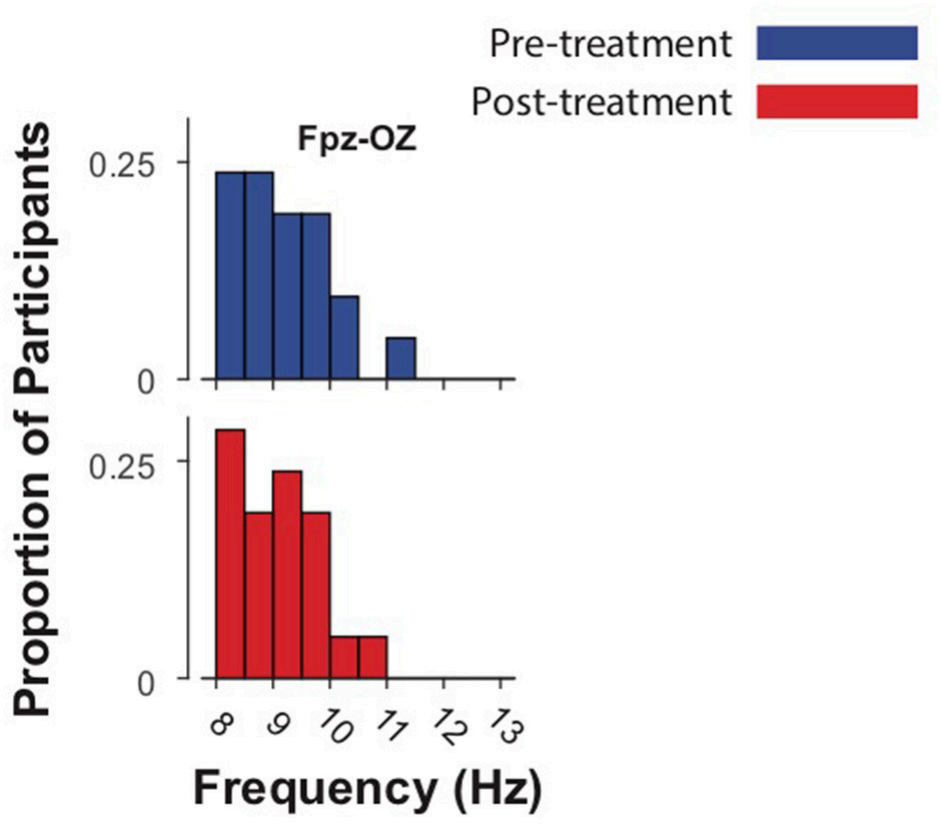
Intrinsic alpha frequencies (IAFs) of 21 subjects in Fpz-Oz electrodes pre- and post-treatment (up to 40 sessions of 5 Hz TMS.) Blue bars represent baseline IAFs. Paired-sample *t*-tests demonstrated no significant difference in IAF before and after treatment.

The mean IAF-PF value at baseline in the Fpz-Oz analysis was 9.21 ± 0.79. The mean IAF-PF values at baseline for the nearest-neighbor electrode pairs 1–8 were 8.42 ± 0.31, 8.46 ± 0.32, 9.02 ± 0.81, 9.11 ± 0.79, 8.65 ± 0.69, 8.81 ± 0.59, 8.98 ± 0.76, 9.14 ± 0.85, respectively. *P*-values for paired *t*-tests comparing IAF at baseline and follow-up did not reach significance in any of the analyses (Figures 1, 2). The third nearest-neighbor electrode did demonstrate increased IAF after treatment (*p* = 0.036), but this was not significant after adjusting for multiple comparisons (corrected *p* > 0.1). Similarly, the mean IAF-CoG did not change after treatment in Fpz-Oz and in nearest-neighbor electrode pairs (Supplementary Figures 1, 2). *P*-values for paired-sample *t*-tests comparing relative alpha power at baseline and follow-up also did not reach significance. We also assessed if there was significant change in EEG relative power across the frequency spectrum (1–30 Hz, 0.5 Hz bins) and found no change (all corrected *p* > 0.1). Finally, there were no significant relationships between baseline IAF and clinical outcomes. For example, the strongest correlations observed remained non-significant: electrode 3 for the PCL-5 (*r* = −0.23, *p* = 0.23) and electrode 8 for the IDSSR (*r* = −0.21, *p* = 0.28). Similarly, non-significant results were obtained when examining baseline relative alpha power and clinical outcomes. See Supplementary Material for a table of all IAF values (for each channel) acquired before and after TMS.

## Discussion

The present EEG analyses detected no change in IAF after a course of 5 Hz TMS for comorbid PTSD and MDD as well as no correlation between baseline IAF and clinical outcomes. Indeed, all of our observed correlations were quite weak and varied among electrodes and clinical outcome type, providing little to no evidence for a relationship between TMS outcomes and baseline IAF. We did not observe any post-treatment EEG changes at the stimulation frequency (5 Hz) or its harmonic (i.e., 10 Hz). This indicates that TMS efficacy, at least in this patient population, does not depend upon a patient's IAF. In other words, IAF lacks predictive utility for TMS treatment response in comorbid MDD and PTSD. This work is somewhat in contrast to prior case studies suggesting a relationship between IAF and clinical outcomes with 10 Hz TMS for MDD (24), although those studies represented a smaller sample size and different clinical population.

Overall, these data provide evidence for the stability of IAF across a course of 5 Hz TMS treatment in a sample of patients with comorbid MDD and PTSD. While interpretations of negative findings must always be pursued with caution, the consistently null results across all analyses performed (and the limited need for multiple comparisons correction to retain these negative results), strongly suggests IAF stability. Therefore, these results support the use of IAF as a target to calibrate future TMS technologies.

Furthermore, based on these results, it is highly unlikely that the mechanism of action for high-frequency TMS involves IAF modification, although we cannot speculate as to whether entrainment occurred briefly during TMS and returned to baseline. However, if MDD and related conditions are conceptualized as thalamocortical dysrhythmias, and IAF is a reflection of this dysrhythmia, then the lack of change in IAF raises important questions for further testing. These results demonstrate the need for acquisition of simultaneous TMS/EEG to evaluate whether brief entrainment occurs, and whether/how those results are related to TMS clinical outcomes.

Limitations of the current study include a lack of structured clinician-rated assessments, modest patient sample size, open-label design and use of a sparse channel EEG. Also, we cannot extrapolate these results to other TMS frequencies. Finally, because our primary analyses on IAF stability required EEG at both baseline and endpoint, participants who did not complete the course of TMS were by definition excluded. It is possible that these participants demonstrated changes in IAF that were therefore unmeasured.

In summary, we found no significant relationship between IAF and TMS in the current study. Future studies may expand on this work by investigating the stability of IAF within different interventions for MDD and PTSD as well as making comparisons of IAF stability across different patient populations.

## Data availability statement

The raw data supporting the conclusions of this manuscript will be made available by the authors, without undue reservation, to any qualified researcher.

## Author contributions

NJP performed the data analyses and wrote the first draft of the manuscript. AZ supervised NJP on data analysis and performed quality control. LLC and NSP designed the parent study (see Funding for further detail). All authors contributed to the manuscript and also read and approved the submitted version.

### Conflict of interest statement

The authors declare that the research was conducted in the absence of any commercial or financial relationships that could be construed as a potential conflict of interest.

## Supplementary material

Supplementary Figure 1 Individual alpha frequencies (IAFs) of 21 subjects in eight EEG channels re-referenced to nearest-neighbor pre- and post-treatment (up to 40 sessions of 5Hz TMS), calculated via the Center of Gravity method (IAF-CoG). Blue bars represent baseline IAF-CoGs. Paired-sample *t*-tests demonstrated no significant difference in IAF before and after treatment.

Supplementary Figure 2 Individual alpha frequencies (IAFs) of 21 subjects in Fpz-Oz electrodes pre- and post-treatment (up to 40 sessions of 5Hz TMS), calculated via the Center of Gravity method (IAF-CoG). Blue bars represent baseline IAF-CoGs. Paired-sample *t*-tests demonstrated no significant difference in IAF before and after treatment.
