# Supplementary material for: Pilot Testing of Peak Alpha Frequency Stability During Repetitive Transcranial Magnetic Stimulation
Source: Front Psychiatry. 2018 Nov 20;9:605. doi: 10.3389/fpsyt.2018.00605 (PMC6256033; doi:10.3389/fpsyt.2018.00605)
Supplement: Supplementary file 1 [file Data_Sheet_1.PDF]

Supplementary Material

Peak Alpha Frequency Stability during Repetitive Transcranial Magnetic Stimulation

Nicholas J. Petrosino[1,2], Amin Zand Vakili[1,2], Linda L. Carpenter[2], and Noah S. Philip[1,2*]

[1]Center for Neurorestoration and Neurotechnology, Providence VA Medical Center
[2]Butler Hospital, Department of Psychiatry and Human Behavior, Alpert Medical School of Brown University, Providence, RI

***Correspondence:**
Noah S. Philip
noah_philip@brown.edu

1 Supplementary Figures and Tables

Mean Intrinsic Alpha Frequencies (IAFs) by EEG Channel Before and After TMS Treatment

**EEG Channel**	**Before TMS**	**After TMS**
Fpz-Oz	9.21 ± 0.79	9.11 ± 0.71
Channel 1: Fp1-Fpz	8.42 ± 0.31	8.51 ± 0.45
Channel 2: Fpz-Fp2	8.46 ± 0.32	8.51 ± 0.42
Channel 3: Fp1-F3	9.02 ± 0.81	8.74 ± 0.58
Channel 4: Fpz-Fz	9.11 ± 0.79	8.88 ± 0.61
Channel 5: F3-Fz	8.65 ± 0.69	8.70 ± 0.57
Channel 6: Fz-Cz	8.81 ± 0.59	8.79 ± 0.62
Channel 7: Cz-Pz	8.98 ± 0.76	8.81 ± 0.63
Channel 8: Pz-Oz	9.14 ± 0.85	9.28 ± 0.72
