# Supplementary figures and images for: Pilot Testing of Peak Alpha Frequency Stability During Repetitive Transcranial Magnetic Stimulation
Source: Front Psychiatry. 2018 Nov 20;9:605. doi: 10.3389/fpsyt.2018.00605 (PMC6256033; doi:10.3389/fpsyt.2018.00605)

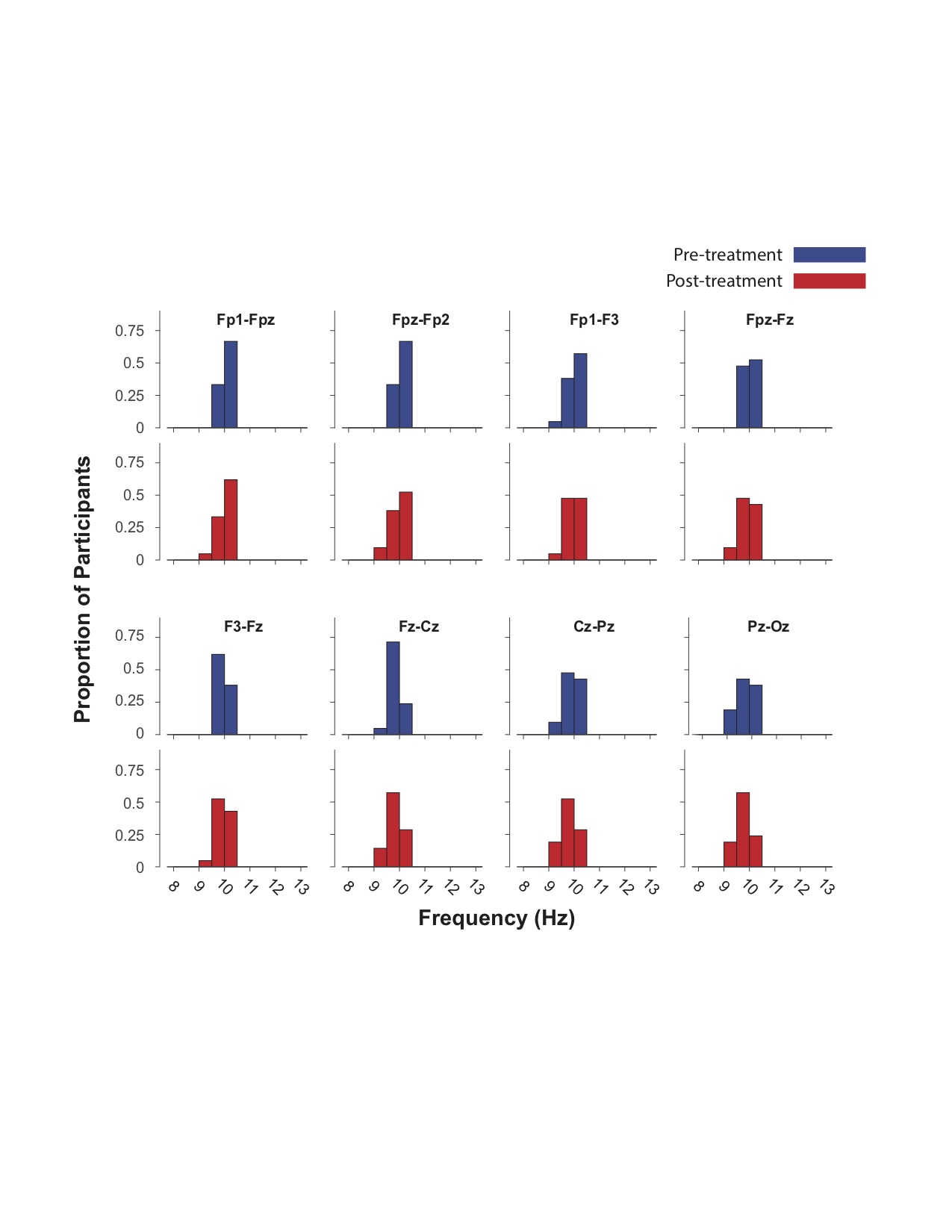

Supplement: Supplementary Figure 1 — Individual alpha frequencies (IAFs) of 21 subjects in eight EEG channels re-referenced to nearest-neighbor pre- and post-treatment (up to 40 sessions of 5Hz TMS), calculated via the Center of Gravity method (IAF-CoG). Blue bars represent baseline IAF-CoGs. Paired-sample t-tests demonstrated no significant difference in IAF before and after treatment. [file Image_1.jpg]

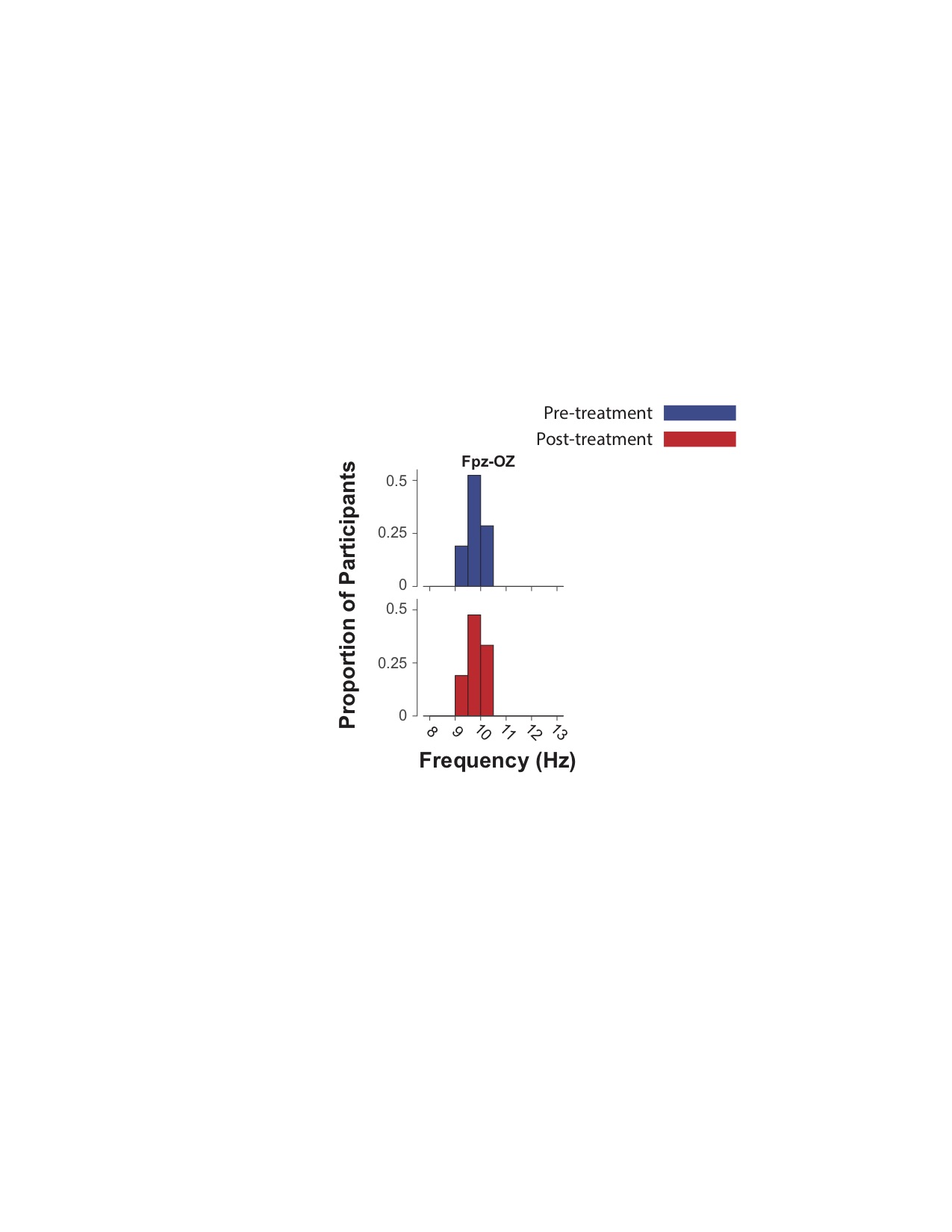

Supplement: Supplementary Figure 2 — Individual alpha frequencies (IAFs) of 21 subjects in Fpz-Oz electrodes pre- and post-treatment (up to 40 sessions of 5Hz TMS), calculated via the Center of Gravity method (IAF-CoG). Blue bars represent baseline IAF-CoGs. Paired-sample t-tests demonstrated no significant difference in IAF before and after treatment. [file Image_2.jpg]
